# Effects of activation of the LINE-1 antisense promoter on the growth of cultured cells

**DOI:** 10.1038/s41598-020-79197-y

**Published:** 2020-12-17

**Authors:** Tomoyuki Honda, Yuki Nishikawa, Kensuke Nishimura, Da Teng, Keiko Takemoto, Keiji Ueda

**Affiliations:** 1 Division of Virology, Department of Microbiology and Immunology, Osaka University Graduate School of Medicine, 2-2 Yamada-oka, Suita, Osaka 565-0871 Japan; 2 Institute for Frontier Life and Medical Sciences, Kyoto University, Kyoto, Japan

**Keywords:** Transposition, Gene expression

## Abstract

Long interspersed element 1 (LINE-1, or L1) is a retrotransposon that constitutes ~ 17% of the human genome. Although ~ 6000 full-length L1s spread throughout the human genome, their biological significance remains undetermined. The L1 5′ untranslated region has bidirectional promoter activity with a sense promoter driving L1 mRNA production and an antisense promoter (ASP) driving the production of L1-gene chimeric RNAs. Here, we stimulated L1 ASP activity using CRISPR-Cas9 technology to evaluate its biological impacts. Activation of the L1 ASP upregulated the expression of L1 ASP-driven ORF0 and enhanced cell growth. Furthermore, the exogenous expression of ORF0 also enhanced cell growth. These results indicate that activation of L1 ASP activity fuels cell growth at least through ORF0 expression. To our knowledge, this is the first report demonstrating the role of the L1 ASP in a biological context. Considering that L1 sequences are desilenced in various tumor cells, our results indicate that activation of the L1 ASP may be a cause of tumor growth; therefore, interfering with L1 ASP activity may be a potential strategy to suppress the growth.

## Introduction

The human genome contains many transposable element-derived sequences, such as endogenous retroviruses and long interspersed element 1 (LINE-1, or L1). L1 is one of the major classes of retrotransposons, and it constitutes ~ 17% of the human genome^1^. Full-length L1 consists of a 5′ untranslated region (UTR), two open reading frames (ORFs) that encode the proteins ORF1p and ORF2p, and a 3′ UTR with a polyadenylation signal. Although most L1s in the genome are truncated and are therefore have no retrotransposition activity^2,3^, some intact L1s are still capable of retrotransposing in the genome. During retrotransposition, L1 RNA is transcribed from the L1 5′ UTR promoter, which is followed by reverse transcription of the L1 mRNA and insertion of the L1 cDNA sequences into the genome^4,5^. L1 retrotransposition requires both ORF1p, an RNA-binding protein with nucleic acid chaperone activity^6^, and ORF2p, a protein with endonuclease and reverse transcriptase (RT) activity^2,3^. Approximately 6000 full-length L1s spread throughout the human genome^7^; however, the biological significance of these widespread L1 sequences remains undemonstrated.

The L1 5′ UTR contains both sense promoter activity, which drives L1 mRNA transcription, and antisense promoter (ASP) activity, which generates L1-gene chimeric transcripts that include neighboring exon sequences^8–10^. Some of these L1-gene chimeric transcripts have been specifically detected in breast and cancer specimens^11^, suggesting a possible role of the L1 ASP in cancer development. Recent advances in transcriptional profiling revealed genome-wide characterization of these L1 ASP-driven L1-gene chimeric transcripts^9,10^. By gene ontology (GO) analysis, Criscione and colleagues showed that these transcripts are involved in diverse cellular processes, including vesicle-mediated transport, intracellular protein transport, mitosis, morphogenesis, and protein modifications^10^. The human-specific L1HS subfamily and the primate-specific L1PA2-8 subfamily contain an ASP-driven ORF named ORF0, which does not share any extensive homology with known genes^12^. ORF0-encoded protein (ORF0p) localizes in close proximity to promyelocytic leukemia protein nuclear bodies (PML-NBs) in the nucleus and stimulates L1 retrotransposition^12^. Since ORF0-proximal exon fusion products are detected in the cells^12^, ORF0-fusion proteins may change their original protein properties and impact some biological processes. Although L1 ASP-driven transcripts, including ORF0 transcripts, are believed to play a role in gene regulation and/or cell signaling, most studies of the L1 ASP transcripts have been gene expression profiling, so this hypothesis currently remains unevaluated by wet experiments.

Here, we developed a novel method to stimulate L1 ASP activity using CRISPR-Cas9 technology and demonstrated that L1 ASP activation enhanced cell growth. We further found that the overexpression of L1 ASP-driven ORF0 also enhanced cell growth. Taken together, L1 ASP stimulation enhances cell growth at least through ORF0 expression. This is the first report demonstrating the role of L1 ASP activity in a biological context.

## Results

### Activation of the L1 ASP using a CRISPR-dCas9-VP64 system in 293T cells

**Figure 1:**
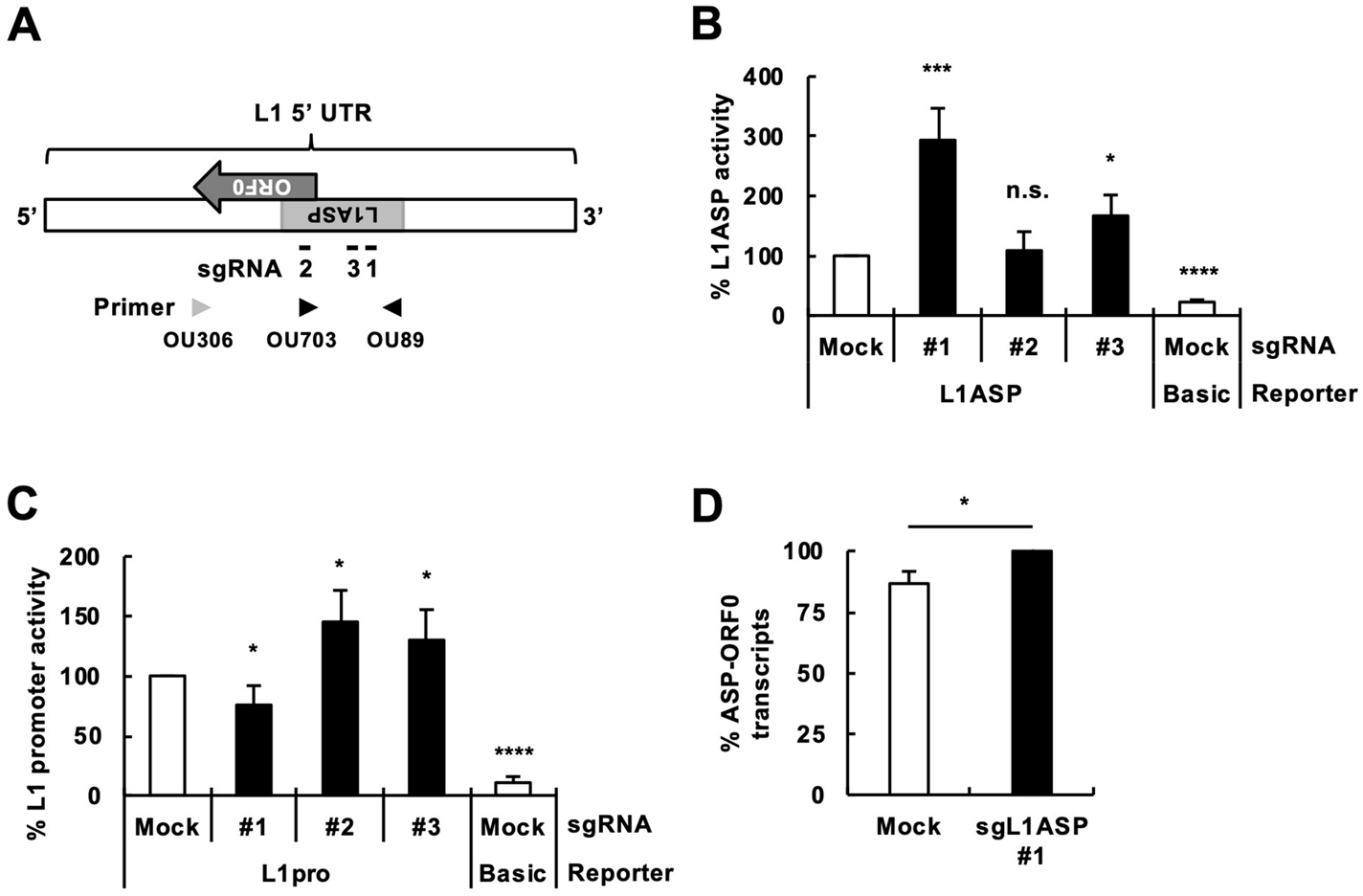
Activation of the L1 antisense promoter (ASP) using a CRISPR-dCas9-VP64 system in 293T cells. (**A**) Schematic representation of the regions in the 5′ UTR of L1_RP_ targeted by guide RNAs, sgL1ASP #1, #2, and #3, (1, 2, and 3, respectively). Arrowheads, primers for real-time RT-PCR specific for L1 ASP-driven ORF0 transcripts (OU306, ORF0-reverse primer; OU89, ASP-forward primer; and OU703, ASP-ORF0-reverse primer). (**B**,**C**) 293T cells were transfected with the expression vectors of dCas9-VP64, an L1 5′ UTR guide RNA, and the L1 ASP (**B**) or promoter (**C**) reporter, together with pCMV-CLuc as a transfection control. Luciferase activity in the culture medium was evaluated at 2 days posttransfection. (**D**) Expression of L1 ASP-driven ORF0 transcritps. Total RNA was extracted from 293T cells expressing dCas9-VP64 and sgL1ASP #1 and reverse transcribed using the OU306 primer. Real-time RT-PCR assays were conducted using the OU89 and OU703 primers. “Mock” of sgRNA represents a mock sgRNA-expressing vector, while “Basic” of Reporter represents a mock reporter. Values are expressed as the means + S.E. of at least four independent experiments. *P < 0.05; ***P < 0.005; ****P < 0.001; and *n.s.* no significance (vs. mock + L1ASP reporter or mock + L1pro reporter in (**B**,**C**)).

To investigate a biological impact of L1 ASP activity, we sought to establish a method to activate L1 ASP. For this purpose, we used dCas9, a Cas9 mutant that binds to a specific DNA sequence but does not cleave it when an sgRNA is coexpressed. dCas9 was fused with VP64, a transcriptional activator (dCas9-VP64), which was then transiently expressed in 293T cells together with sgRNAs targeting the ASP of L1_RP_ (sgL1ASP #1–#3 in Fig. 1A). Then, we evaluated the L1 promoter and ASP activity using luciferase assays. When dCas9-VP64 was expressed with sgL1ASP #1 or #3, the L1 ASP was activated, as expected (Fig. 1B). On the other hand, sgL1ASP #2 did not affect L1 ASP activity (Fig. 1B). We next evaluated the effect of these sgRNAs on L1 promoter activity. When dCas9-VP64 was expressed with sgL1ASP #2 or #3, the L1 promoter was slightly activated (Fig. 1C). On the other hand, sgL1ASP #1 suppressed L1 promoter activity (Fig. 1C). sgL1ASP may produce alternative RNA species, which could affect translation of the reporter. To directly evaluate the ASP activity, we analyzed the amount of reporter mRNAs by real-time RT-PCR. The amount of the reporter mRNA was increased by sgL1ASPs #1 and #3, which was consistent with the results obtained by luciferase assay (Figs. S1, 1). Because we wanted to modulate L1 ASP activity, we focused on sgL1ASP #1 and #3 for further analyses. To further confirm L1 ASP activation by sgL1ASPs, we expressed dCas9-VP64 together with sgL1ASPs in HeLa and OL cells. sgL1ASP #1 enhanced L1 ASP activity in both cell lines, which was consistent with the result in 293 T cells (Figs. S2, 1). sgL1ASP #3 also enhanced L1 ASP activity in HeLa cells but not in OL cells (Fig. S2). These results showed that dCas9-VP64 in the presence of a specific sgL1ASP can activate the L1 ASP with different amplitudes (~ 300% for sgL1ASP #1 and < 200% for #3) and with different effects on the L1 promoter. We next sought to detect upregulation of endogenous L1 ASP-driven gene transcripts upon our L1 ASP stimulation. The L1 ASP reportedly upregulates the expression of L1 ORF0, whose product stimulates L1 retrotransposition^12,13^. Real-time RT-PCR specific for L1 ASP-driven ORF0 transcripts revealed that the transcripts were indeed upregulated in sgL1ASP-expressing cells (Fig. 1D). We also detected enhanced L1 retrotransposition in sgL1ASP-expressing cells (Fig. S3)^14^, further supporting the idea that our system stimulates endogenous L1 ASPs and upregulates ORF0 expression.

### Transcriptome analysis of L1 ASP activation

To screen the biological consequence of L1 ASP activation, we compared the transcriptomes of cells coexpressing dCas9-VP64 with mock, sgL1ASP #1, or #3. Based on the results shown in Fig. 1, we reasoned that genes related to L1 ASP activation would be upregulated in both sgL1ASP #1- and #3-treated cells. In contrast, those related to the L1 5′ UTR promoter would be upregulated in sgL1ASP #1-treated cells but downregulated in sgL1ASP #3-treated cells or vice versa. Furthermore, because the effect of sgL1ASP #1 on L1 ASP activity was stronger than that of sgL1ASP #3 (Fig. 1), the amplitude of gene expression change was expected to be more robust in sgL1ASP #1-treated cells. Based on these assumptions, we searched for genes related to L1 ASP activation using RNA-seq. We found 230 genes upregulated by L1 ASP activation (Table S1). Although we did not detect L1 ASP-gene chimeric reads likely because of low-coverage short-read sequencing, we indeed detected upregulation of 13 previously reported L1 ASP-related genes in our list (genes in bold in Table S1)^10^. GO analysis of these genes (see "Materials and methods" section) revealed that genes related to L1 ASP activation were associated with the regulation of cell cycle (the Bonferroni adjusted p-value < 0.005). We evaluated the expression of three representative genes, i.e., *CLASP1*, *MAPK12*, and *CHFR*, in the regulation of cell cycle pathway using real-time RT-PCR and confirmed that their expression was indeed affected in sgL1ASP-treated cells (Fig. S4). These results suggest that L1 ASP activation may be involved in cell cycle regulation and thereby cell growth. For further analyses, we used sgL1ASP #1 because of its strong L1 ASP activation.

### Effects of L1 ASP activation on cell growth

**Figure 2:**
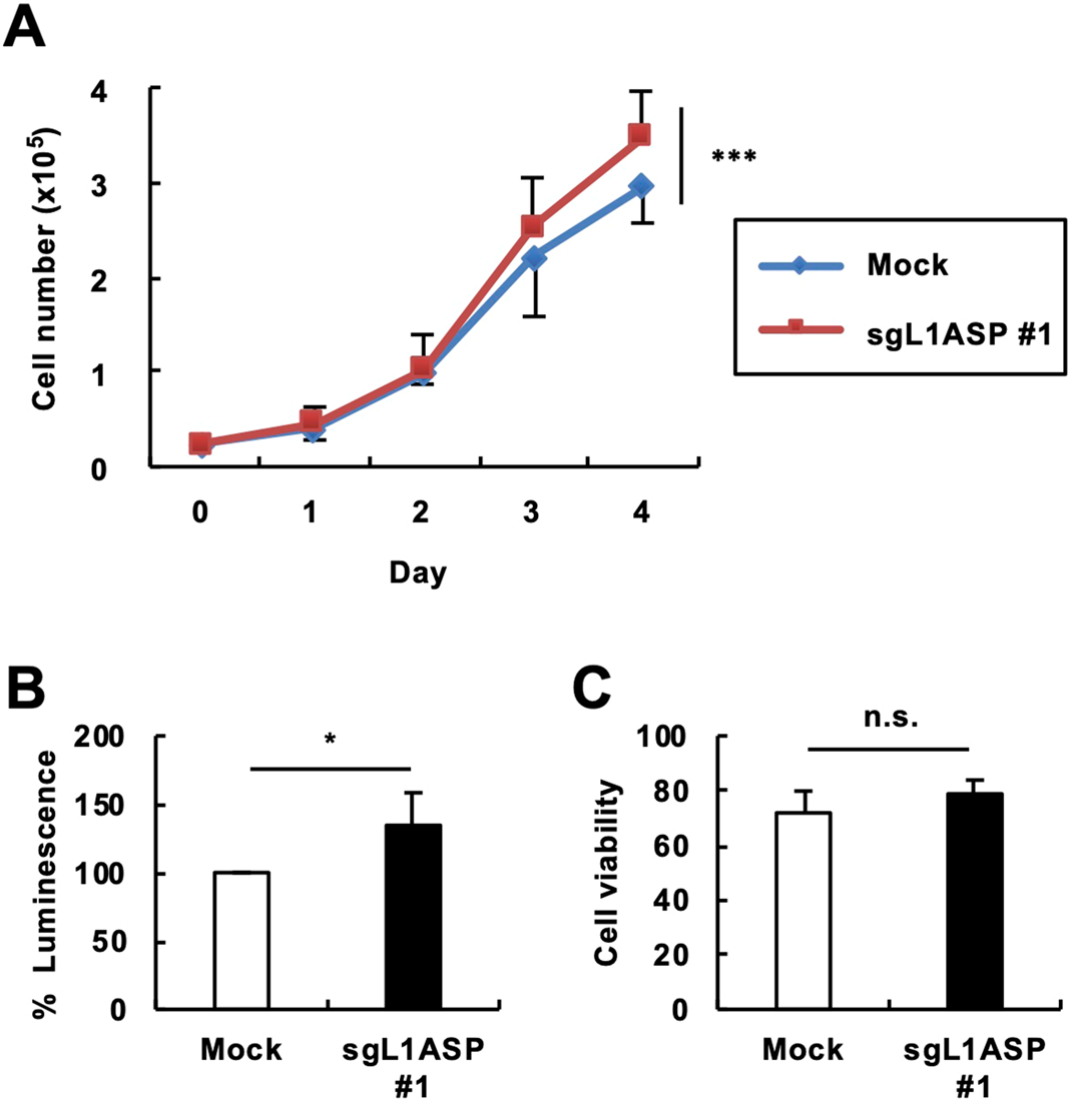
Effects of L1 ASP activation on cell growth. 293T cells were transfected with the expression vectors of dCas9-VP64 and sgL1ASP #1. (**A**) Growth kinetics. The cell numbers were counted every day. (**B**) The viable cell numbers at 4 days posttransfection were evaluated using the the CellTiter-Glo Luminescent Cell Viability Assay kit. (**C**) The cell viabilities were evaluated by trypan blue staining. Values are expressed as the means of at least five independent experiments. The error bars indicate S.E. of the independent experiments. *P < 0.05; ***P < 0.005; *n.s.* no significance.

To evaluate the effect of L1 ASP activation on cell growth, we examined the growth kinetics of 293T cells expressing dCas9-VP64 with or without sgL1ASP #1 (Fig. 2A). We found that 293T cells expressing dCas9-VP64 and sgL1ASP #1 grew faster than those expressing dCas9-VP64 and mock sgRNA (Fig. 2A). Evaluation of cell proliferation using the CellTiter-Glo Luminescent Cell Viability Assay kit confirmed the results shown in Fig. 2A (Fig. 2B). Furthermore, trypan blue staining revealed that cell viability was comparable between sgL1ASP #1- and mock sgRNA-expressing cells (Fig. 2C), excluding the possible contribution of apoptosis to cell number difference. These results suggest that activation of the L1 ASP can stimulate cell growth.

### Cell cycle analysis upon L1 ASP activation

**Figure 3:**
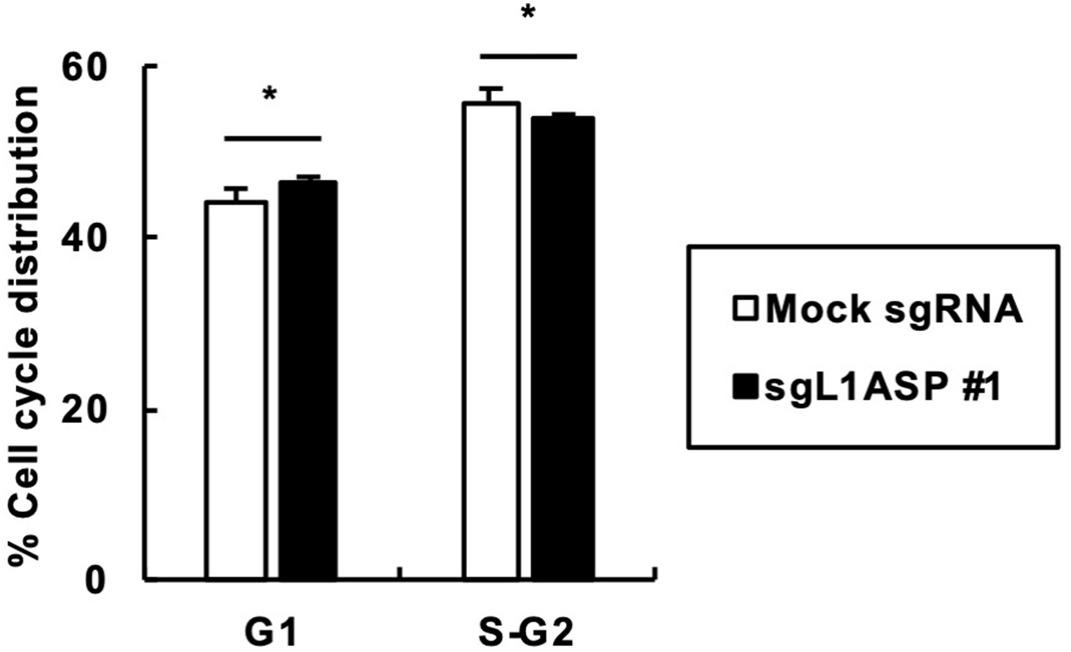
Cell cycle analysis upon L1 ASP activation. 293T cells were transfected with the expression vectors of dCas9-VP64 and sgL1ASP #1. The transfected cells were stained with propidium iodide and analyzed by flow cytometry. Values are expressed as the means + S.E. of five independent experiments. *P < 0.05 (vs. mock sgRNA-expressing cells).

To gain more insights into the effect of L1 ASP activation on cell growth, we conducted cell cycle analysis. We found that the cells in the S-G2 phase was descreased by sgL1ASP #1 expression, while those in the G1 phase was increased (Fig. 3). These results are consistent with the GO analysis of genes differentially expressed by L1 ASP activation. Together with the results shown in Fig. 2, L1 ASP activation likely enhances cell growth through shortening the duration of the S-G2 phase.

### Effects of ORF0 expression on cell growth

**Figure 4:**
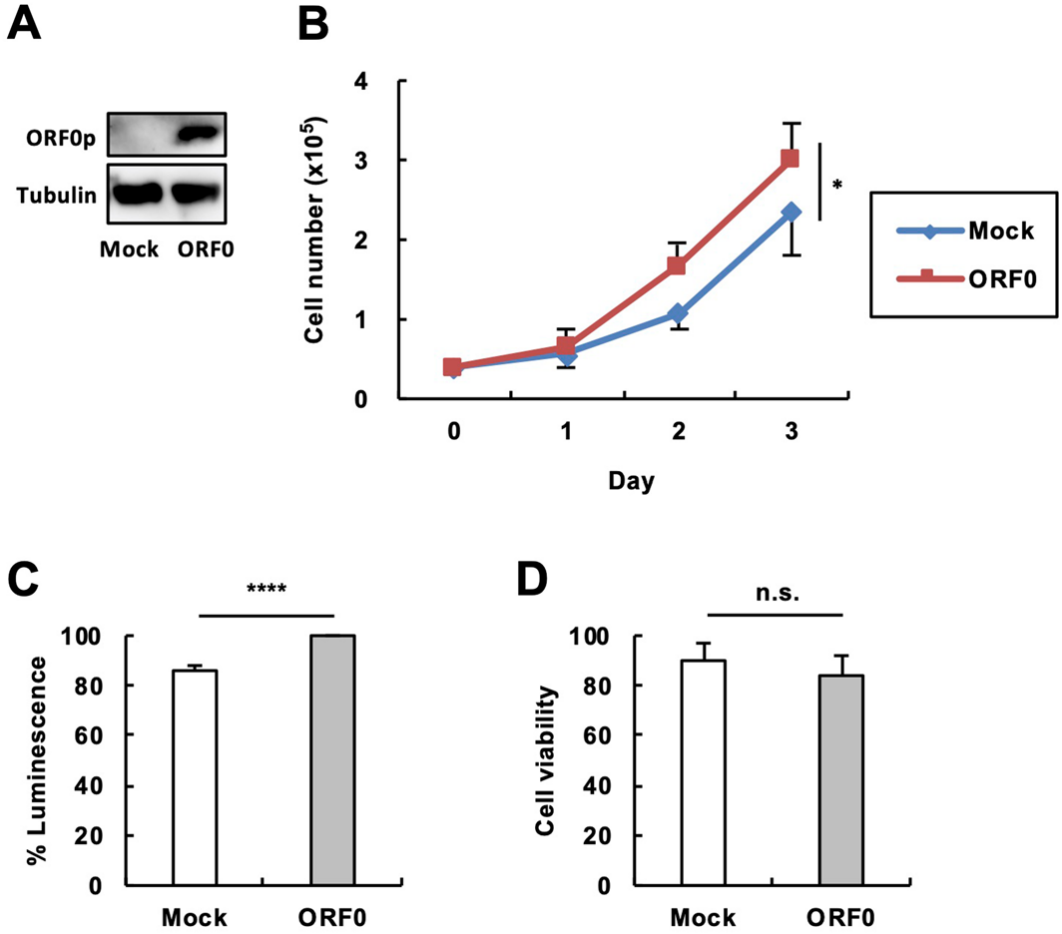
Effects of ORF0 expression on cell growth. 293T cells were transfected with the expression vectors of ORF0. (**A**) Expression of ORF0p in the transected cells. The expression of ORF0p in the cell homogenate was evaluated by western blot using an anti-FLAG (for ORF0p) or an anti-tubulin (for tubulin) antibody. A vector expressing only the One-Strep-FLAG tag was used as a mock vector. (**B**) Growth kinetics. The cell numbers were counted every day. (**C**) The viable cell numbers at 2 days posttransfection were evaluated using the the CellTiter-Glo Luminescent Cell Viability Assay kit. (**D**) The cell viabilities were evaluated by trypan blue staining. Values are expressed as the means of at least three independent experiments. The error bars indicate S.E. of the independent experiments. *P < 0.05; ****P < 0.001; *n.s.* no significance.

To further investigate the mechanism of how the L1 ASP regulates cell growth, we noticed L1 ASP-driven L1 ORF0 since we detected upregulation of these transcripts upon our L1 ASP stimulation (Fig. 1D). Thus, we reasoned that L1 ASP activation increases ASP-driven ORF0 expression, which contributes to enhancement of cell growth. To evaluate this possibility, we overexpressed ORF0p (Fig. 4A) and investigated the effect on cell growth. As expected, 293T cells expressing ORF0p grew faster than mock cells (Fig. 4B). Evaluation of cell proliferation using the CellTiter-Glo Luminescent Cell Viability Assay kit confirmed the results shown in Fig. 4B (Fig. 4C). Furthermore, trypan blue staining revealed that cell viability was comparable between ORF0p-expressing and mock cells (Fig. 4D), excluding the possible contribution of apoptosis to cell number difference. These results are consistent with the effect of L1 ASP activation demonstrated in Fig. 2 and suggest that L1 ASP activity fuels cell growth at least through L1 ASP-driven ORF0 expression.

## Discussion

L1 sequences occupy ~ 17% of the human genome^1^. Considering that they contain several elements for transcription and posttranscriptional modifications^15^, understanding the biological impacts of the L1 sequences is important. Among the elements, the L1 ASP is known to regulate the expression of L1-gene chimeric transcripts^8–10^; however, its impacts in a biological context remain undetermined. In this study, we investigated the biological impacts of L1 ASP activation. To this aim, we developed a novel tool, i.e., a CRISPR-Cas9-based L1 ASP activation system. We successfully activated the L1 ASPs by expressing sgL1ASP and dCas9-VP64 (Fig. 1, Figs. S1, S2). Using a CRISPR-Cas9-based L1 ASP activation system, we demonstrated that L1 ASP activation stimulated cell growth and cell cycle progression (Figs. 2, 3). Furthermore, the overexpression of ASP-driven ORF0 also enhanced cell growth (Fig. 4). Collectively, our results revealed a biological impact of the L1 ASP, i.e., stimulating cell growth. Our results also highlight the usefulness of our strategy to stimulate L1 ASP activity, which is a powerful tool that will enable future investigations to understand the significance of the L1 ASPs.

Expression from L1 sequences is usually silenced in somatic cells because dysregulated L1 retrotransposition may impair genome integrity^16^. Similarly, expression of endogenous viral elements is also epigenetically silenced because they contain several regulatory elements for transcription and posttranscriptional modifications, whose dysregulation can induce transcriptome changes^17,18^. Since L1 ASP activity is also limited by DNA methylation in normal tissues, inhibition of DNA methylation by 5-aza-cytidine induces expression of L1 ASP-driven transcripts^11^. On the other hand, desilencing of L1 sequences has been frequently reported in various types of tumor cells^19^. Because of this, it is speculated that desilencing of L1 sequences contributes to tumorigenesis. The activation of the L1 promoter and ASP in tumor cells is thought to stimulate L1 retrotransposition and thereby increase the likelihood of oncogenic mutagenesis. Consistently, some oncogenic chemicals, such as 2-amino-1-methyl-6-phenylimidazo[4,5-b]pyridine and 2-amino-3,8-dimethyl-imidazo[4,5-f]quinoxaline, can induce L1 retrotransposition^20^. Conversely, capsaicin, which exhibits anticancer and/or growth-inhibition effect in various cancers^21,22^, suppresses L1 retrotransposition^23^. Our results suggest another possibility that activation of the L1 ASP by desilencing L1 sequences can stimulate tumor cell growth. Thus, desilencing of the L1 sequences stimulates oncogenic processes in multiple ways, i.e., at least through activating L1 retrotransposition and the L1 ASPs, which highlights the importance of the control of L1s in tumor prevention.

At present, the precise mechanisms of how the L1 ASPs regulate cell growth are unclear. The L1 ASPs directly drive the expression of ORF0 transcripts (Fig. 1D) and directly or indirectly drive the expression of genes related to the cell cycle and/or cell proliferation (Table S1), which results in enhanced cell growth (Fig. 2). Indeed, a previous GO analysis of L1 ASP-driven transcripts found their involvement in mitosis^10^. Consistently, our RNA-seq revealed that genes related to the regulation of the cell cycle were upregulated by L1 ASP activation. For examples, CLASP1 and CHFR are reportedly associated with microtubule and spindle dynamics^24,25^, and downregulation of MAPK12 suppresses cell proliferation^26^. Since CHFR is one of L1 ASP transcripts reported previously^10^, L1 ASP activation may directly drive CHFR expression and indirectly induce CLASP1 and MAPK12 expressions, thereby accelerate cell cycle progression. In this study, we demonstrated a contribution of ORF0p to enhancement of cell growth (Fig. 4). The human- and primate-specific L1 ASPs reportedly drive ORF0-containing transcripts, whose encoded protein, ORF0p, is predominantly nuclear and localizes in close proximity to PML-NBs^12^. PML-NBs are implicated in various biological processes, including mitosis^27^. Because our cell cycle analysis demonstrated that L1 ASP activation shortens the duration of the S-G2 phase, L1 ASP activation may accelerate mitosis through PML-NBs and L1 ASP-driven ORF0p, thereby supporting efficient cell growth.

In conclusion, we suggested a possible role of the L1 ASP in cell growth. To our knowledge, this is the first study to demonstrate the significance of L1 ASP activity in a biological context. Since the L1 sequences are desilenced in various tumor cells^11,28,29^, activation of the L1 ASP may contribute to tumor growth. In that sense, our findings suggest that the control of L1 ASP activity by small compounds represents a novel strategy to modulate tumor growth potential.

## Materials and methods

### Cells

293T cells (a human embryonic kidney cell line from ATCC), HeLa cells (a human cervical epithelial cell line from ATCC), and OL cells (a human oligodendroglioma cell line^30^) were cultured in Dulbecco’s modified Eagle’s medium (DMEM, Nakalai, Japan) supplemented with 5%, 10%, and 5% fetal bovine serum (FBS), respectively.

### Plasmids

The luciferase-based L1 retrotransposition reporter plasmid, pYX014, was kindly provided by Dr. Wenfeng An (South Dakota State University, USA)^14^. The human L1 promoter activity reporter plasmid, pGLuc-5′-UTR, was generated previously (accession number for 5′ UTR: AF148856)^23^. The reporter plasmid for L1 ASP activity, pGLuc-L1-ASP, was generated by subcloning the ASP of L1, which belongs to the L1HS subfamily, from the pYX014 plasmid into a pGLuc-Basic plasmid (New England BioLabs, Ipswich, MA). A parental pGLuc-Basic plasmid was used as a mock reporter. For plasmids expressing sgRNAs against L1 ASP (sgL1ASP #1–#3), a pair of oligos (#1, 5′-TTT CTT GGC TTT ATA TAT CTT GTG GAA AGG ACG AAA CAC CGA GGT GGA GCC TAC AGA GGC-3′ and 5′-GAC TAG CCT TAT TTT AAC TTG CTA TTT CTA GCT CTA AAA CGC CTC TGT AGG CTC CAC CTC-3′; #2, 5′-TTT CTT GGC TTT ATA TAT CTT GTG GAA AGG ACG AAA CAC CGC AAG GCG GCA ACG AGG CTG-3′ and 5′-GAC TAG CCT TAT TTT AAC TTG CTA TTT CTA GCT CTA AAA CCA GCC TCG TTG CCG CCT TGC-3′; #3, 5′-TTT CTT GGC TTT ATA TAT CTT GTG GAA AGG ACG AAA CAC CGT GGA GCC CAC CAC AGC TCA-3′ and 5′-GAC TAG CCT TAT TTT AAC TTG CTA TTT CTA GCT CTA AAA CTG AGC TGT GGT GGG CTC CAC-3′) were annealed and inserted into the *Afl*II sites of a gRNA_Cloning Vector (Addgene #41824). These oligos were designed using the CRISPRdirect server (http://crispr.dbcls.jp/) and the L1 ASP sequence as a query. The L1 ASP sequence was divided into three parts (5′, middle, 3′) and a designed guide RNA was chosen from each part. A parental gRNA_Cloning Vector was used as a plasmid expressing mock sgRNA. The expression plasmid of dCas9-VP64, pcDNA-Cas9m4-VP64, was provided through Addgene (#47319). For the ORF0 expression, One-Strep-FLAG-tagged ORF0 was cloned into the pCAG plasmid similarly to the previous study^13^. The pCAG plasmid expressing only the One-Strep-FLAG tag was used as a mock vector.

### L1 promoter and ASP assays

L1 promoter and ASP assays were conducted as described^23^ with some modifications. Briefly, 293T cells were cotransfected with pGLuc-5′-UTR, pCMV-CLuc (New England BioLabs), and pcDNA-Cas9m4-VP64, together with a plasmid expressing mock sgRNA or sgL1ASP using Lipofectamine 2000 reagent (Invitrogen, Carlsbad, CA). At 2 days after transfection, the *Gaussia* and *Cypridina* luciferase activities were measured using *Gaussia* or *Cypridina* Luciferase Assay Kits (New England BioLabs) according to the manufacturer’s instructions. The *Gaussia* luciferase (GLuc) activity was normalized to the corresponding *Cypridina* luciferase (CLuc) activity.

### L1 retrotransposition assay

The L1 retrotransposition assay was conducted as previously described^23^. Briefly, 293T cells were transfected with pYX014 and pcDNA-Cas9m4-VP64, together with a plasmid expressing mock sgRNA or sgL1ASP using Lipofectamine 2000 reagent. At 4 days after transfection, *Firefly* and *Renilla* luciferase activities were measured using a Dual-Luciferase Reporter Assay System (Promega, Madison, WI) according to the manufacturer’s instructions; readings were collected with a single-well luminometer (Berthold, Lumat LB 9507, Bad Wildbad, Germany). *Renilla* luciferase was constitutively expressed from the reporter construct and used for normalization of transfection efficiency.

### RNA-seq analysis

RNA-seq analysis was conducted as previously described^31^. Total RNA was extracted from 293T cells expressing dCas9-VP64 together with mock, sgL1ASP #1, or sgL1ASP #3 using an miRNeasy kit (Qiagen, Hilden, Germany). Library preparation was performed using a TruSeq stranded mRNA sample prep kit (Illumina, San Diego, CA) according to the manufacturer’s instructions. Sequencing was performed on an Illumina HiSeq 2500 platform in 75-base single-end mode. Sequenced reads were mapped to the human reference genome sequences (hg19) using TopHat v2.0.13 in combination with Bowtie2 ver. 2.2.3 and SAMtools ver. 0.1.19. The fragments per kilobase of exon per million mapped fragments (FPKMs) were calculated using Cuffdiff version 2.2.1 with a strand specific mode (-library-type fr-firststrand). A total of 230 genes were upregulated by > 1.5-fold (SgL1ASP #1 to #3 and sgL1ASP #3 to mock) (see Table S1). GO analysis of differentially expressed genes was conducted using the DAVID server (https://david.ncifcrf.gov/) and the significance was evaluated by the Bonferroni adjusted p-value. Access to raw data concerning this study was submitted under Gene Expression Omnibus (GEO) accession number GSE152634.

### Real-time RT-PCR

Real-time RT-PCR was performed as previously described^23^. Total RNA was extracted from the indicated cells using TRI Reagent (Sigma-Aldrich, St. Louis, MO) and was reverse transcribed using a Verso cDNA Synthesis Kit (Thermo Fisher Scientific, Waltham, MA). Quantitative real-time RT-PCR assays were carried out using Fast SYBR Green Master Mix (Thermo Fisher Scientific) and gene-specific primers with a QuantStudio 6 (Thermo Fisher Scientific). HPRT1 mRNA was quantified and used to standardize the total amount of cDNA. For real-time RT-PCR specific for ASP-driven ORF0 transcripts, cDNA was synthesized by reverse transcription using the ORF0-reverse primer (OU306) and RT-PCR assays were conducted using the ASP-forward (OU89) and ASP-ORF0-reverse primers (OU703). The gene-specific primers used in this study are as follows:

- GLuc-forward primer, 5′-AGA GAT GGA AGC CAA TGC CC-3′,
- GLuc-reverse primer, 5′-CAG ATC GAC CTG TGC GAT GA-3′,
- CLuc-forward primer, 5′-CTG TGA TCT GAC CCC CAA CC-3′,
- CLuc-reverse primer, 5′-CTG TTG TCC CCT CAG GCA AT-3′,
- OU306 primer, 5′-CAC GGA TCC TCA AAG AAA GGG GTG ACG GAC G-3′,
- OU89 primer, 5′-GTG GAA TTC CTG CAG AGG TTA CTG CTG TC-3′,
- OU703 primer, 5′-GGG GGA GGG GCG CCC GCC AT-3′,
- CLASP1-forward primer, 5′-AAG CAA TAC GAT TGG CCG GA-3′,
- CLASP1-reverse primer, 5′-GTG GGG ATG GAG TTA GGC TG-3′,
- MAPK12-forward primer, 5′-ACC CTG GAT GAC TTC ACG GA-3′,
- MAPK12-reverse primer, 5′-GGC AGC GTG GAT ATA CCT CAG-3′,
- CHFR-forward primer, 5′-CTC CTC CGC TCT CGT GTT G-3′,
- CHFR-reverse primer, 5′-GTT GTG GCT TCC CAG CAT TG-3′,
- HPRT1-forward primer (HPSF-F)^32^, 5′-GGA CTA ATT ATG GAC AGG ACT G-3′, and,
- HPRT1-reverse primer (HPSF-R)^32^, 5′-GCT CTT CAG TCT GAT AAA ATC TAC-3’.

### Cell growth assay

293 T cells were transfected with plasmids expressing sgL1ASP and dCas9-VP64 or that expressing ORF0 using Lipofectamine 2000 reagent. At 24 h after transfection, the cells stimulated by CRISPR-Cas9-based system (2.5 × 10^4^ cells/well) were replated into a 24-well plate. For the ORF0 expression experiment, 293T cells (4 × 10^4^ cells/well) were seeded in a 24-well plate. The cell number was manually counted every day and the cell viability was evaluated by trypan blue staining. The cell proliferation was also evaluated using the CellTiter-Glo Luminescent Cell Viability Assay (Promega) according to the manufacturer’s instructions. The values of the sgL1ASP- or ORF0-expressing wells were normalized to those of the mock-expressing wells.

### Cell cycle analysis

Transfected 293T cells were stained with propidium iodide using the Cell Cycle Phase Determination Kit (Cayman Chemical, Ann Arbor, MI) according to the manufacturer’s instructions. The stained cells were analyzed with a CytoFLEX (Beckman Coulter, CA) and Kaluza analysis software (Beckman Coulter).

### Western blot analysis

Western blot was performed as previously described^33^ with some modifications. Briefly, the cell homogenate was subjected to SDS-PAGE and transferred onto polyvinylidene difluoride membrane (Bio-Rad Laboratories, Hercules, CA). The membrane was then blotted with a mouse anti-FLAG (Thermo Fisher Scientific) or a mouse anti-tubulin (Wako Pure Chemical Industries, Osaka, Japan) antibody. After three washes, a horseradish peroxidase-conjugated secondary antibody was applied. The bound antibodies were detected using a Clarity Western ECL Substrate (Bio-Rad Laboratories).

### Statistics

Statistical significance was assessed using a two-tailed Student’s *t*-test with a threshold of p < 0.05. Other statistical assessment was used, when indicated.

## Supplementary Information

Supplementary Figures.

Supplementary Table S1.

## References

[1] Lander ES (2001). Initial sequencing and analysis of the human genome. Nature.

[2] Beck CR (2010). LINE-1 retrotransposition activity in human genomes. Cell.

[3] Brouha B (2003). Hot L1s account for the bulk of retrotransposition in the human population. Proc. Natl. Acad. Sci. U.S.A..

[4] Burns KH, Boeke JD (2012). Human transposon tectonics. Cell.

[5] Feng Q, Moran JV, Kazazian HH, Boeke JD (1996). Human L1 retrotransposon encodes a conserved endonuclease required for retrotransposition. Cell.

[6] Khazina E (2011). Trimeric structure and flexibility of the L1ORF1 protein in human L1 retrotransposition. Nat. Struct. Mol. Biol..

[7] Penzkofer T, Dandekar T, Zemojtel T (2004). L1Base: from functional annotation to prediction of active LINE-1 elements. Nucleic Acids Res..

[8] Speek M (2001). Antisense promoter of human L1 retrotransposon drives transcription of adjacent cellular genes. Mol. Cell. Biol..

[9] Ishiguro K, Higashino S, Hirakawa H, Sato S, Aizawa Y (2018). Establishment of a genome-wide and quantitative protocol for assessment of transcriptional activity at human retrotransposon L1 antisense promoters. Genes Genet. Syst..

[10] Criscione SW (2016). Genome-wide characterization of human L1 antisense promoter-driven transcripts. BMC Genomics.

[11] Cruickshanks HA, Tufarelli C (2009). Isolation of cancer-specific chimeric transcripts induced by hypomethylation of the LINE-1 antisense promoter. Genomics.

[12] Denli AM (2015). Primate-specific ORF0 contributes to retrotransposon-mediated diversity. Cell.

[13] Nakayama R, Ueno Y, Ueda K, Honda T (2019). Latent infection with Kaposi’s sarcoma-associated herpesvirus enhances retrotransposition of long interspersed element-1. Oncogene.

[14] Xie Y, Rosser JM, Thompson TL, Boeke JD, An W (2011). Characterization of L1 retrotransposition with high-throughput dual-luciferase assays. Nucleic Acids Res..

[15] Singer T, McConnell MJ, Marchetto MCN, Coufal NG, Gage FH (2010). LINE-1 retrotransposons: mediators of somatic variation in neuronal genomes?. Trends Neurosci..

[16] Honda T (2016). Links between human LINE-1 retrotransposons and hepatitis virus-related hepatocellular carcinoma. Front. Chem..

[17] Sofuku K, Parrish NF, Honda T, Tomonaga K (2015). Transcription profiling demonstrates epigenetic control of non-retroviral RNA virus-derived elements in the human genome. Cell Rep..

[18] Szpakowski S (2009). Loss of epigenetic silencing in tumors preferentially affects primate-specific retroelements. Gene.

[19] Rodić N (2014). Long interspersed element-1 protein expression is a hallmark of many human cancers. Am. J. Pathol..

[20] Okudaira N (2013). Long interspersed element-1 is differentially regulated by food-borne carcinogens via the aryl hydrocarbon receptor. Oncogene.

[21] Chapa-Oliver AM, Mejía-Teniente L (2016). Capsaicin: from plants to a cancer-suppressing agent. Molecules.

[22] Friedman JR (2017). Anti-cancer activity of natural and synthetic capsaicin analogs. J. Pharmacol. Exp. Ther..

[23] Nishikawa Y (2018). Inhibition of LINE-1 retrotransposition by capsaicin. Int. J. Mol. Sci..

[24] Maiato H (2003). Human CLASP1 is an outer kinetochore component that regulates spindle microtubule dynamics. Cell.

[25] Burgess A (2008). Chfr interacts and colocalizes with TCTP to the mitotic spindle. Oncogene.

[26] Cui C, Shi X (2017). miR-187 inhibits tumor growth and invasion by directly targeting MAPK12 in osteosarcoma. Exp. Ther. Med..

[27] Lång A, Lång E, Bøe SO (2019). PML bodies in mitosis. Cells.

[28] Takai D, Yagi Y, Habib N, Sugimura T, Ushijima T (2000). Hypomethylation of LINE1 retrotransposon in human hepatocellular carcinomas, but not in surrounding liver cirrhosis. Jpn. J. Clin. Oncol..

[29] Hur K (2014). Hypomethylation of long interspersed nuclear element-1 (LINE-1) leads to activation of proto-oncogenes in human colorectal cancer metastasis. Gut.

[30] Nakamura Y (2000). Isolation of Borna disease virus from human brain tissue. J. Virol..

[31] Nagasawa M (2020). Long non-coding RNA MANCR is a target of BET bromodomain protein BRD4 and plays a critical role in cellular migration and invasion abilities of prostate cancer. Biochem. Biophys. Res. Commun..

[32] Valadan R (2015). Data supporting the design and evaluation of a universal primer pair for pseudogene-free amplification of HPRT1 in real-time PCR. Data Br..

[33] Honda T (2016). Long-term expression of miRNA for RNA interference using a novel vector system based on a negative-strand RNA virus. Sci. Rep..

