# Supplementary material for: Effects of activation of the LINE-1 antisense promoter on the growth of cultured cells
Source: Sci Rep. 2020 Dec 17;10:22136. doi: 10.1038/s41598-020-79197-y (PMC7746726; doi:10.1038/s41598-020-79197-y)
Supplement: Supplementary file 1 — Supplementary Figures. [file 41598_2020_79197_MOESM1_ESM.pdf]

Supplemental Information

Effects of activation of the LINE-1 antisense promoter on the growth of cultured cells

Tomoyuki Honda, Yuki Nishikawa, Kensuke Nishimura, Da Teng, Keiko Takemoto, and Keiji Ueda

Supplemental Figure S1-S5.

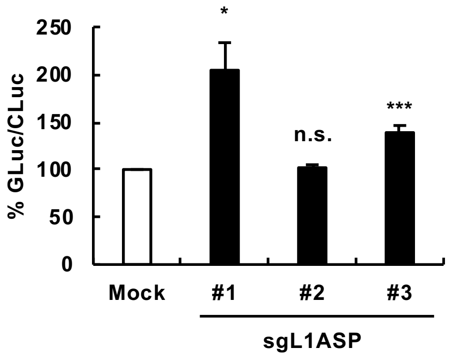

Supplemental Figure S1

Evaluation of L1 ASP activation by real-time RT-PCR. 293T cells were transfected with the expression vectors of dCas9-VP64, an L1 5' UTR guide RNA, sgL1ASP, and the L1 ASP reporter, together with pCMV-CLuc as a transfection control. At 2 days posttransfection, total RNA was extracted from the cells and subjected to real-time RT-PCR analysis using the GLuc- and CLuc-specific primers. The amount of GLuc mRNA was normalized to the corresponding amount of CLuc mRNA. sgL1ASP #1, #2, and #3 targeted different sites of the L1 5' UTR as indicated in Figure 1A. "Mock" represents a mock sgRNA-expressing vector. Values are expressed as the means + S.E. of three independent experiments (mock was set to 100%). *, $P<0.05$; ***, $P<0.005$; n.s., no significance.

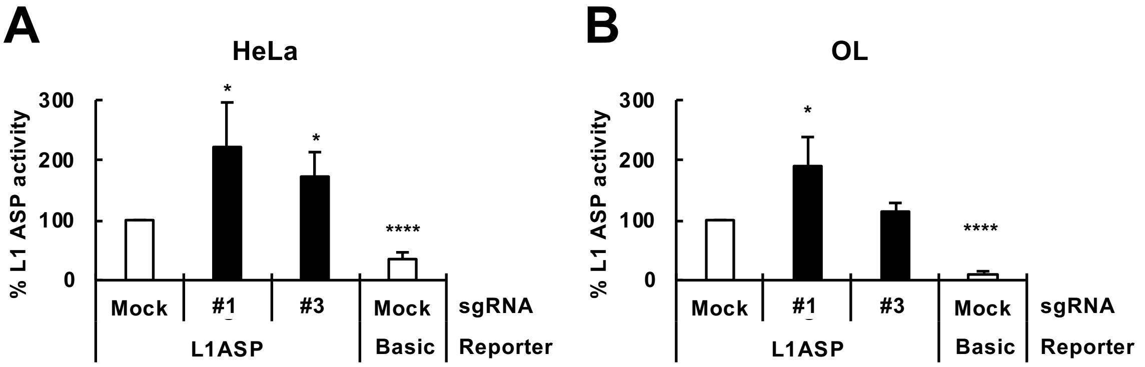

Supplemental Figure S2

Activation of the L1 ASP using a CRISPR-Cas9-VP64 system in HeLa and OL cells. HeLa (A) or OL (B) cells were transfected with the expression vectors of dCas9-VP64, sgL1ASP, and the L1 ASP reporter, together with pCMV-CLuc as a transfection control. Luciferase activity in the culture medium was evaluated at 2 days posttransfection. sgL1ASP #1 and #3 targeted different sites of the L1 5' UTR as indicated in Figure 1A. "Mock" of sgRNA represents a mock sgRNA-expressing vector, while "Basic" of Reporter represents a mock reporter. Values are expressed as the means + S.E. of at least five independent experiments. *, $P<0.05$; ****, $P<0.001$.

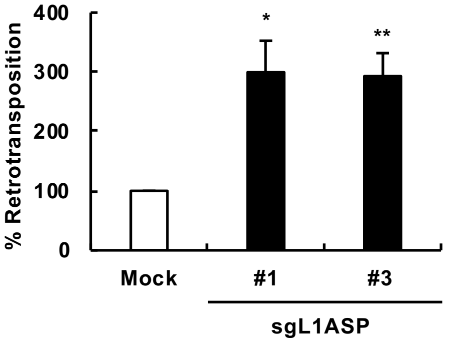

Supplemental Figure S3

The effect of L1ASP activation on L1 retrotransposition. 293T cells were transfected with the L1 retrotransposition reporter construct together with dCas9-VP64 and sgL1ASPs. Luciferase activity was evaluated at 4 days posttransfection. sgL1ASP #1 and #3 targeted different sites of the L1 5' UTR as indicated in Figure 1A. "Mock" represents a mock sgRNA-expressing vector. Values are expressed as the means + S.E. of three independent experiments. *, $P<0.05$; **, $P<0.01$.

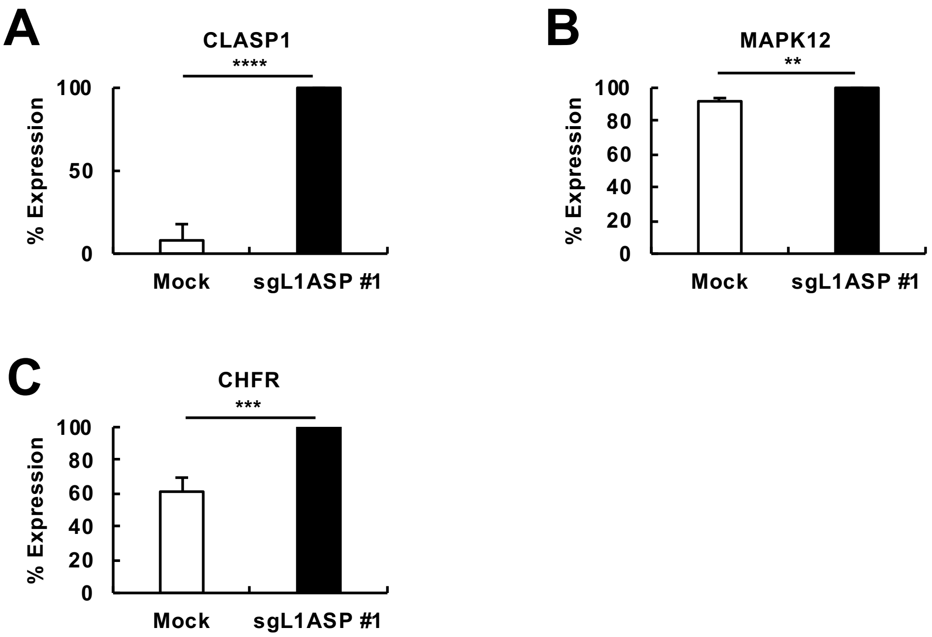

Supplemental Figure S4

Evaluation of the expression of CLASP1, MAPK12, and CHFR after L1 ASP activation. 293T cells were transfected with the expression vectors of dCas9-VP64 and sgL1ASP #1. Total RNA was extracted from the transfected cells and subjected to real-time RT-PCR using the CLASP1- (A), MAPK12- (B), and CHFR- (C) specific primers. The expression was normalized to that of HPRT1. Values are expressed as the means+ S.E. of three independent experiments (sgL1ASP #1 was set to 100%). **, P<0.01; ***, P<0.005; ****, P<0.001.

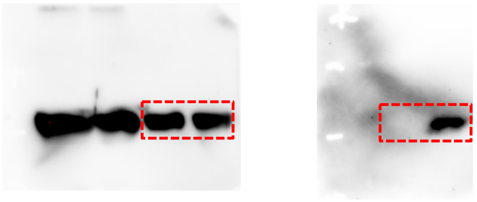

Supplemental Figure S5
Original full-length blots before cropping. The original blots for Figure 4A.
